# NaDES Application in Cosmetic and Pharmaceutical Fields: An Overview

**DOI:** 10.3390/gels10020107

**Published:** 2024-01-28

**Authors:** Carla Villa, Debora Caviglia, Francesco Saverio Robustelli della Cuna, Guendalina Zuccari, Eleonora Russo

**Affiliations:** 1 Department of Pharmacy, Section of Drug and Cosmetic Chemistry, Viale Benedetto XV 3, 16132 Genoa, Italy; carla.villa@unige.it (C.V.); debora.caviglia@edu.unige.it (D.C.); guendalina.zuccari@unige.it (G.Z.); 2 Environmental Research Center, ICS Maugeri SPA SB, Institute of Pavia, IRCCS, Via Maugeri 2, 27100 Pavia, Italy; fsaveriorobustelli@unipv.it

**Keywords:** natural deep eutectic solvents, NaDES and formulations, NaDES and drug delivery, NaDES and cosmetic, bioactive compound extraction

## Abstract

Natural deep eutectic solvents (NaDES) represent a new generation of green, non-flammable solvents, useful as an efficient alternative to the well-known ionic liquids. They can be easily prepared and exhibit unexpected solubilizing power for lipophilic molecules, although those of a hydrophilic nature are mostly used. For their unique properties, they can be recommend for different cosmetic and pharmaceutical applications, ranging from sustainable extraction, obtaining ready-to-use ingredients, to the development of biocompatible drug delivery responsive systems. In the biomedical field, NaDES can be used as biopolymer modifiers, acting as delivery compounds also known as “therapeutic deep eutectic systems”, being able to solubilize and stabilize different chemical and galenical formulations. The aim of this review is to give an overview of the current knowledge regarding natural deep eutectic solvents specifically applied in the cosmetic and pharmaceutical fields. The work could help to disclose new opportunities and challenges for their implementation not only as green alternative solvents but also as potential useful pathways to deliver bioactive ingredients in innovative formulations.

## 1. Introduction

Currently, interest in the development of sustainable processes and green bioactive compounds from renewable sources is steadily increasing in the cosmetic and pharmaceutical fields. From an extractive point of view, avoiding unfriendly solvents, saving sources and energy, and recycling waste have become primary objectives for the pharmaceutic and cosmetic context, according to the green extraction principles [1]. Conventional organic solvents are commonly used for extracting aromas, perfumes, medicines, and dyes from plants, but they are often not sustainable due to toxicity, high environmental impact and flammability [2]. For this reason, in recent years, research in the green extraction context has focused its attention on new non-toxic, biodegradable green solvents [3].

In this context, ionic liquids (ILs) and deep eutectic solvents (DES) can represent an excellent alternative to conventional hazardous organic solvents [2]. ILs are defined as salts deriving from the combination of an organic cation and an anion, characterized by a melting point below 100 °C, being in most cases liquids at room temperature [4]. DESs are defined as homogeneous eutectic mixtures obtained by mixing two or more pure components (liquids or solids, ions or neutral molecules) acting as hydrogen bond acceptors (HBA) and hydrogen bond donors (HBD) [5]. High thermal stability, low volatility, and wide ranges of viscosity and polarity are some of the most interesting properties belonging to both ILs and DES [6,7]. In particular, the class of IL organic salts is characterized by low melting point and minimal vapor pressure, and they can be modified in terms of polarity and selectivity for different applications such as chemical or enzymatic reactions [8,9]. Unfortunately, their use is restricted due to their high toxicity and high production costs, including those for synthesis, purification and disposal [10]. These limitations can be overcome by deep eutectic solvents with comparable or better physical properties and phase behaviors than ILs [11]. First introduced by Abbott et al. [12], DES represent a great and successful alternative to ILs, characterized by easy preparation, purity and low costs [13]. The process to obtain DES involves the simple mixing of a hydrogen-bond acceptor (HBA) (like a quaternary ammonium salt) and a hydrogen-bond donor (HBD) at a suitable molar ratio [14]. Their interaction gives rise to supramolecular compounds, with peculiar chemical-physical characteristics [15,16], with a charge delocalization that is responsible for the lowering of the mixture melting temperature with respect to the individual components (generally from room temperature to 70 °C) [7]. Unfortunately, the use of DES, like green solvents at room temperature, can be hindered by their melting points being too high [17]. 

In this regard, a new generation of greener DES of natural origin has emerged over the past decade. In nature, it has been hypothesized that in plants, different metabolites can form eutectic mixtures which play different biological roles. They can act as an alternatives to water and lipids, with the ability to transport water-insoluble compounds inside the cells, explaining the co-presence of water soluble and insoluble compounds in the botanical matrix. For this reason, when these metabolites (i.e., sugars, alcohols, amino acids, organic acids) form DES, they are called “Natural DES” (NaDES) [18]. Their green properties and behaviors were first described by Choi et al. in 2011 [17,18,19]. 

**Figure 1:**
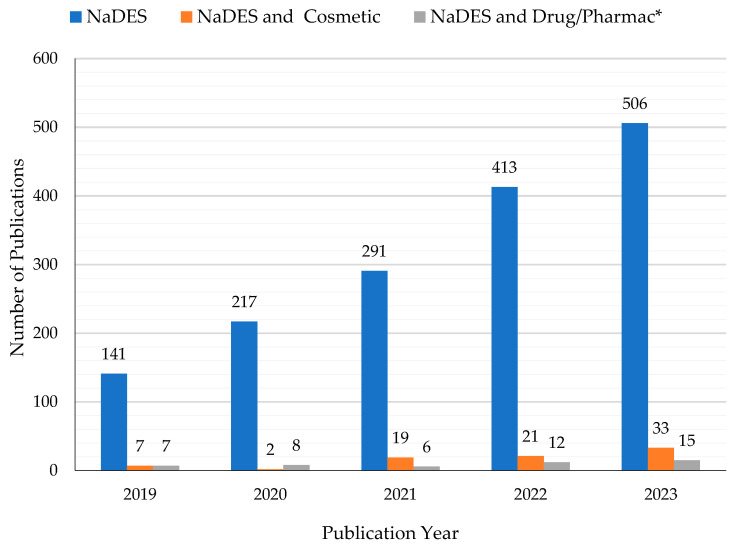
Histogram showing the increase in publications in the past 5 years (2019–2023) regarding the keywords “NaDES or natural deep eutectic solvent” and the corresponding small number related to “NaDES and Cosmetic” and “NaDES and Drug or Pharmac*” (data available on Scopus accessed on 20 December 2023).

Synthetic NaDES can reproduce this natural behavior, and they are considered promising new green solvents to be applied in several fields, such as in the cosmetic, pharmaceuticals and food areas. NADES were used successfully to extract phenolic compounds from plant material. For example, in recent years, research has been carried out on the NaDES extraction of phlorotannins from the brown alga *Fucus vesiculosus* L. [20,21]. Phlorotannins are polyphenols with antioxidant, anti-inflammatory, antiallergic, antibacterial, and antitumor properties. They have a wide range of cosmetic applications, e.g., in sunscreens as anti-aging and UV-protective agents, and in in food packaging films as preservatives. Some authors have suggested the use of NaDES as solvents to stabilize proteins (lysozyme, amylase, photosynthetic enzymes) and DNA [18,22,23]. This opportunity led to an increasing interest in NaDES as drug delivery systems for active, but poorly soluble, ingredients [24,25,26]. NaDES show a wide polarity range and high solubilization strength for a variety of compounds. They present several advantages over classical solvents, ILs, and DES, such as natural origin, low cost, biodegradability, absence of toxicity, sustainability, and simple preparation [22]. Although NaDES are recognized as being slightly toxic and with a low environmental impact, it must be mentioned that they show the phenomenon of eutrophication [27]. As extractive alternative solvents, NaDES allow the achievement of efficient extractions when compared to conventional solvents [28,29,30,31]. Moreover, they often improve the stability and storage of the extracted compounds of interest, such as phenols, β-carotene, and α-tocopherol [22,23,29,32,33]. Despite the myriad of research fields in which NaDES are involved for diverse types of applications, the dermocosmetic and pharmaceutical topics are relatively unexplored, as can be seen from Figure 1.

In the last 5 years (2019–2023), more than 1600 papers have been published on this subject. Most of them deal with the use of these eutectic systems for the extraction of bioactive compounds from botanical matrices and/or agrifood waste, for the most varied applications. In order to narrow and detail the object of the work, this review considers and reports the latest research and results limited to cosmetic and pharmaceutical applications, where NaDES are explicitly included in the formulations. Papers in which the cosmetic and/or pharmaceutical potential is only mentioned and NaDES are proposed as alternative extractive solvents are summarized and cited in Table 1.

### 1.1. NaDES Preparation

Many NaDES mixtures are biodegradable and have low toxicity [55,56,57], partially due to their natural origin. Most of their components present an intrinsic cosmetic or pharmaceutical activity, being well-known and used ingredients (organic acids, sugars, alcohols and polyols, amino acids and quaternary ammonium salts). 

Particularly from a cosmetic point of view, this aspect presents many advantages: increasing the naturality of the compositions and the concentration of active ingredients, stabilizing them without adding preservatives, reducing the number of ingredients and allowing a synergistic effect to improve the biological activity of the formulation.

The preparation of NaDES yields easy results with high purity and no waste formation [58] according to the fundamental principles of green chemistry [59].

As mentioned above, NaDES can be prepared by mixing an HBA (i.e., choline chloride, choline acetate or betaine) with an HBD (glycerol, urea, glucose, sorbitol, fructose, etc.), with or without water [17], mainly applying these most common and different physical methods:

1. Heating and stirring method [17], where two components are mixed with a magnetic stirring bar, in a 50 °C water bath until a clear viscous liquid is formed, about 30–90 min later [17,22,60,61]. Otherwise, it is possible to follow the conditions stated by Abbot et al. 2003 [12], or heating at 80 °C under continuous stirring [60,62,63].
2. Freeze-drying method [64], which is the least used and based on freeze-drying by sublimation of both the NaDES aqueous portion and the individual components of the NaDES. This method makes it possible to achieve pure NaDES.
3. Evaporation method [17], which involves the use of rotary evaporator to allow the components’evaporation and dissolution in water at 50 °C. The liquid that is obtained is transferred to a silica gel desiccator until it reaches a constant weight.
4. Grinding method, where the component mixture is ground in a mortar with a pestle, at room temperature, until formation of a homogeneous liquid [65].
5. Ultrasound-assisted heating method, where the component mixture is exposed to ultrasonication until a homogeneous liquid is formed [66].
6. Microwave irradiation technique, where the mixture is irradiated in a microwave oven at low power emission and for a few seconds [67].

**Figure 2:**
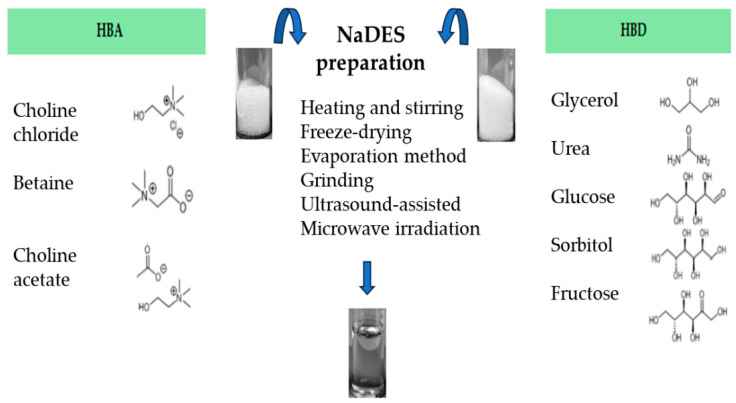
Different preparation methods to obtain NaDES.

The methods mentioned above are shown in Figure 2. The microwave-assisted preparation of NaDES represents a promising green technique, due to its advantages such as higher yields, lower energy consumption and shorter reaction times [68].

### 1.2. NaDES Structure

The structure and properties of NaDES are conferred by the type and ratio of components and also by the hydrogen (H) bonds established between the metabolites themselves [17,69,70]. The H bonds’strength is related to the phase-transition temperature, stability and solvent properties of the mixture [64]; their key roles in important NaDES features and behaviors (such as stability and formation) depend on their number and location [17].

The lowering of the mixture melting temperature, with respect to the single components, is due to the formation of a charge delocalization between the HBA and HBD and to the van der Waals forces that allow blocking the crystallization of the compounds [29]. Usually, a low freezing point can be determined by a higher binding capacity between the HBD and HBA [70].

The NaDES structures have been evaluated through nuclear magnetic resonance spectroscopy (NMR) studies, crystallographic data, fast atom bombardment-mass spectrometry (FAB)-MS and Fourier transform infrared spectroscopy (FT-IR) [17,60,71]. Thanks to the nuclear Overhauser effect spectroscopy (NOESY) spectra obtained, it has been seen that NaDES are characterized by a supramolecular structure mainly due to bonds established between HBAs and HBDs [17]. This supramolecular structure changes after water dilution [59]; in fact, it was observed that the presence or absence of water plays a significant role. This behavior occurs because the H bond systems that NaDES are able to form, will gradually fade when diluted with water, until disappearing when the water amount exceeds 50% v/v. In this regard, it has been observed that the degradation of concentrated NaDES was slower than that of diluted ones [72].

The amount of added water tolerated by the eutectic system should be determined for each NaDES. Moreover, the types of components used to form NaDES can influence their physicochemical properties, such as viscosity, conductivity, density, and polarity [60].

Craveiro et al. have demonstrated that water can increase polarity, which affects the solubility of NaDES [73]. Simultaneously, dilution with water can result in a decrease in viscosity. This rheological behavior is one of the main problems that NaDES present [17]. A high viscosity interferes with the flow of substances and decreases the extraction efficiency [74]; this problem can be overcome by heating. The high temperature and thermal expansion lead to increased molecular force and to structural damage, respectively [75]. Another way to reduce the viscosity is dilution with water, since, as already mentioned, water leads to the breaking of the hydrogen bonds and consequently to a lower viscosity [60].

**Figure 3:**
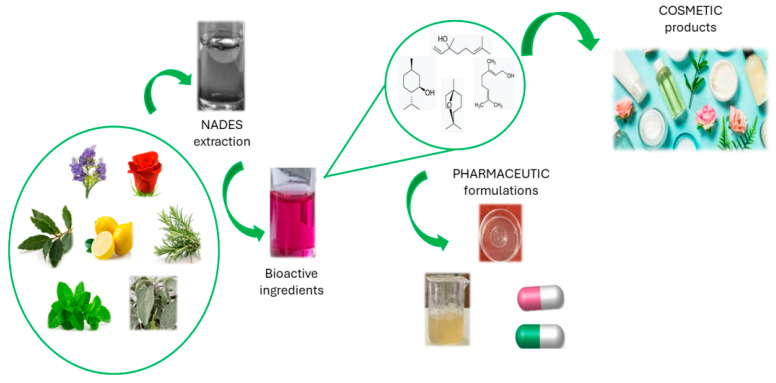
Use of NaDES in cosmetic and pharmaceutical fields.

Several works in the literature describe the use of NaDES to obtain, from natural sources, bioactive compounds that can be used in cosmetic and pharmaceutical formulations (Figure 3). The main advantage consists of the possibility of directly adding the NaDES-based extract itself to all types of topical formulations, both in the cosmetic and pharmaceutical fields, without dramatic changes in the rheological properties or sensorial profile [76]. However, only a few can actually be used for cosmetic applications because of safety or regulatory issues [75].

In the section below, some papers dealing with the use of NaDES to obtain active principles with excellent properties that can be exploited in the future have been taken into consideration.

## 2. NaDES in the Cosmetic Field

Jeong et al. [30] developed an extractive procedure to obtain catechins from *Camellia sinensis* (*C. sinensis*) green tea leaves, including epigallocatechin-3-gallate (EGCG), a powerful antioxidant. Their optimized extraction method involved the use of a ternary DES mixture, suitable for both pharmaceutical topical preparations and cosmetic formulations. The authors prepared NaDES by both the heating [17] and freeze-drying [64] methods, selecting glycerol, xylitol, citric acid, betaine, D-(+)-glucose, D-sorbitol, D-(+)-maltose, maltitol, urea, D-(−)-fructose, D-(+)-galactose, and sucrose in an adequate molar ratio. They tested several extractive methods, including ultrasound-assisted extraction (UAE), agitation, heating, and heating with agitation. 

All of the green solvents assessed by the authors allowed for very efficient extractions, but taking into account the limits of use in cosmetic formulations and the production costs, the final choice was a NaDES composed of betaine, glycerol and D-(+)-glucose, 4:20:1 (BGG-4). Compared to conventional solvent extractions (water and organic solvents), this mixture allowed for a better extraction of EGCG and improved stability. The best extraction conditions, identified by the response surface methodology, involved the application of UAE at room temperature for 6.5 min, using 81% BGG-4. In conclusion, it was highlighted that BGG-4 is an excellent extractive solvent and stabilizer for catechins of *C. sinensis*, useful in topical formulations.

Also, Vasyliev et al. [77] indicated NaDES as promising solvents to extract antioxidant bioactive compounds for use in cosmetic formulations. In their work, they applied the UAE method with one NaDES based on choline chloride (as the HBA) to extract polyphenols from waste tomato pomace. To enhance extraction efficiency, they prepared the NaDES in the presence of water. In particular, they tested a mixture composed of choline chloride: 1,2-propanediol (1:2 *v*/*w*): water (10% *w*/*w*) (DESs-1) and another one containing choline chloride: lactic acid (1:2 *v*/*w*): water (10% *w*/*w*) (DESs-2). 

The tomato pomace extracts obtained were then characterized and used as antioxidant agents (being rich in phenolic acids and flavanols) to develop a natural cream formulation (oil-in-water emulsion). The main phenolic compounds extracted with DESs-1 and DESs-2 were gallic acid, chlorogenic acid, caffeic acid, trans-cinnamic acid, p-coumaric acid and ferulic acid. The extract obtained with DESs-2 afforded higher amounts of quercetin, caffeic and ferulic acid, displaying an enhanced antioxidant power when compared to DESs-1. The cosmetic formulation also containing DESs-2 showed greater antioxidant activity. Both emulsions, stabilized by DES, demonstrated antifungal activity against *Candida albicans*. In conclusion, this study showed that DES can extract polyphenols from agri-food waste, such as tomato pomace, to be used as antioxidant additives in the cosmetics industry.

The research team of Petkov et al. [78] also investigated NaDES as possible solvents for extracting bioactive compounds from natural sources. The authors evaluated the antioxidant activity of extracts from *Plantago major* (*P. major*), *Sideritis scardica* (*S. scardica*) and propolis obtained by UAE, assessing the extraction efficiency in terms of total phenols and flavonoids content, using 10 different NaDES. Betaine-malic acid-water 1:1:6 (BMAH), citric acid-1,2-propanediol 1:4 (CAPD), lactic acid-fructose 5:1 (LAFr), lactic acid-1,2-propanediol 1:1 (LAPD), choline chloride-glucose-water 5: 2:25 (XXGlH), choline chloride-glycerol 1:1 (XXGly), choline chloride-1,2-propanediol 1:3 (XXPD), and choline chloride1,2-propanediol-water 1:1:1 (XXPDH) were prepared by the heating and stirring method. In contrast, choline chloride-urea 1:1 (XXU) and choline chloride-xylitol 4:1 (XXXy) were obtained by the vacuum evaporation method The most effective phenolic extraction for both *P. major* and *S. scardica* was achieved using XXGlH as the solvent. When compared to EtOH 70%, used as a reference, XXPDH, XXPD and LAPD extracted a greater phenolic amount and the same quantity of flavonoids. Moreover, in contrast to *P. major* and *S. scardica*, propolis showed a strong correlation between phenolic concentration and antioxidant capacity. In conclusion, Petkov et al. asserted that NaDES extracts can be directly incorporated into formulations considering their intrinsic properties, such as biocompatibility, low toxicity, and excellent antioxidant activity.

Another natural bioactive compound, namely Naringerin (NA), a flavonoid already utilized in various formulations, was extracted by El Maaiden et al. [79] from dried aerial parts of *Searsia tripartita* (*ST*), using NaDES. This study focused on six eutectic solvents, prepared by the heating technique [80], composed by choline chloride in a 1:2 ratio with formic acid (DES-1), ethylene glycol (DES-2), lactic acid (DES-3), urea (DES-4), and glycerol (DES-5), and in a 2:1 ratio with citric acid (DES-6). These solvents were, therefore, used for the NA extraction by UAE from *ST* powder. After characterization, DES-1 was chosen for further analyses as the best-performing solvent with the highest NA concentration, while DES-6 showed the lowest yields. El Maaiden et al. presented the best operative conditions based on their achievements, involving an extraction time of 10 min at 50 °C, with an ultrasound amplitude of 75 W and a solid–liquid ratio of 1/60 g/mL. These extracts proved to be excellent antiaging agents for their antioxidant activity and as enzyme inhibitors of tyrosinase, collagenase, elastase, and hyaluronidase, which are responsible for skin aging.

Further, Jamaleddine et al. [81] conducted an extractive study using NaDES on tomato pomace (TP), rich in bioactive compounds. Specifically, they prepared and used four kinds of NaDES to extract TP by UAE. Jamaleddine and coworkers proposed a novel strategy for sustainable formulations by incorporating their extraction medium directly into the formulations. They selected four different methods, present in the literature, for preparing their NaDES. DES 1, composed of glycerol:glucose (1:3) and water (30%), was prepared using the method developed by Wils et al. [82]; DES 2 (DL-menthol-lactic acid 8:1) was obtained by the method of Silva et al. [83]; DES 3, composed of lactic acid-glucose (5:1) and 15% water, was prepared following the method of Fernandez et al. [84], and finally, DES 4 (L-proline-glycerol 1:2.5 and 30% water) was prepared according to Wu et al. [85] with some modifications. TP extractions required a matrix maceration in DES 1 for 2 h at 40 °C (ratio NaDES/TP 32:2 *w*/*w*) and for 30 min at 50 °C with DES (NaDES/TP 20:3.4 *w*/*w*). For DES 3 and DES 4, the UAE procedure was carried out for 1 h at 40 °C, with a solid–liquid ratio of 40/2 *v*/*w* and 20/2 *v*/*w*, respectively. The results showed that DES 1 demonstrated great suitability for the extraction of phenolic acids, flavones, flavonols and tannins. DES 2 could extract carotenoids, lipids and tocopherol. DES 3 demonstrated greater efficiency for phenols, while flavones, flavanols and flavanones were ultimately extracted by DES 4. Finally, the extracts obtained were employed to develop four cosmetic formulations: a peel-off mask (containing DES 1); a lip balm (DES 2); a water-soluble mask (DES 3); and a moisturizing cream (DES 4).

Another research team, Jin et al. [86], produced extracts with excellent skin properties using a mixture of dried and ground leaves of *Ginkgo biloba* L (GB), *Cinnamomum camphora* (L) *J. Presl* (CC), and *Cryptomeria japonica* (L.f.) *D. Don* (CJ). Using safe, stable, and cost-effective substances approved by the European Commission (2006) [87], Jin et al. prepared and assessed 15 different cosmetics-compliant NaDES using the heating method [17,88]: glycerol-xylitol 2:1 (DES 1), glycerol-maltose 3:1 (DES 2), glycerol-sorbitol 2:1 (DES 3), glycerol-fructose 3:1 (DES 4), glycerol-sucrose 3:1 (DES 5),glycerol-glucose 3:1 (DES 6), glycerol-maltitol 3:1 (DES 7), glycerol-malic acid 1:1 and 1:2 (DES 8 and DES 9), lactic acid-glucose 1:2 (DES 10), fructose-sucrose 1:1 (DES 11), fructose-sucrose-glucose 1:1:1 (DES 12), betaine-sucrose 1:1 and 1:2 (DES 13 and DES 14) and 1:1 betaine:glucose (DES 15). This approach facilitated the safe development of an ISO extraction technique capable of producing a significant quantity of extracts that can be directly incorporated into cosmetic formulations. DES 1 was selected as the most efficient solvent for the extraction of isoquercetin (ISO yield 861 μg/g), found in GB, CC and CJ leaf extracts. Finally, using the central composite design approach, Jin et al. collected data on specific bioactivities with several leaf extract mixtures and identified the best-performing in terms of increasing antioxidant activity and anti-tyrosinase and anti-elastase effects.

The study by Hsieh et al. [89] highlighted the potential of natural DES as alternative solvents to volatile organic solvents (VOS), with the same or even better efficiency. In particular, the authors extracted gingerols from *Zingiber officinale Roscoe* (ginger) powder to obtain extracts that could be incorporated directly into formulations, without the need for work-up steps for product isolation. NaDES were designed and prepared by the ultrasonication assisted method, with three different hydrogen bond acceptors (choline chloride, betaine and L-carnitine) and five polyalcohols (triethylene glycol, ethylene glycol, 1,3-propanediol, glycerol, and 1,3-butanediol) as hydrogen bond donors in opportune molar ratios: choline chloride-triethylene glycol 1:4 (CC-TriG), choline chloride-ethylene glycol 1:2 (CC-EG), choline chloride-1,3-propanediol 1:4 (CC-PG), choline chloride-glycerol 1:2 (CC-gly), choline chloride-1,3-butanediol 1:4 (CC-ButG), betaine-triethylene glycol 1:4 (Bet-TriG), betaine-ethylene glycol 1:2 (Bet-EG), betaine-1,3-propanediol 1:4 (Bet-PG), betaine-glycerol 1:2 (Bet-gly), betaine-1,3-butanediol 1:4 (Bet-ButG), L-carnitine-triethylene glycol 1:4 (Lcat-TriG), L-carnitine-ethylene glycol 1:2 (Lcat-EG), L-carnitine-1,3-propanediol 1:4 (Lcat-PG), L-carnitine-glycerol 1:2 (Lcat-gly), and L-carnitine-1,3-butanediol 1:4 (Lcat-ButG). Ultrasonication-assisted extractions were carried out after diluting all of the NaDES samples with 75% water v/v, reducing viscosities for a more effective extraction. Three NaDES (Bet-ButG, Lcat-Trig and Lcat-ButG) resulted in the most efficient eutectic systems, containing the highest concentration of gingerols. Finally, the authors suggested the best operative conditions for UAE (50 °C for 30 min with a 30/1 solvent/solid ratio *v*/*w*) that could maintain the antioxidant activity of gingerols and prevent phenol degradation.

Rocha et al. [90] emphasized the effectiveness of NaDES-based extracts from botanical matrices as cosmetic ingredients. By the heating and stirring technique, the research team prepared three eutectic solvents composed as follows: lactic acid-glycerol 1:1 and 10% water (NADES 1), lactic acid-glycine 5:1 and 13% water (NADES 2), and lactic acid-sodium citrate 4:1 and 31% water (NADES 3). NADES 1–3 were subjected to an accurate physicochemical characterization (melting point, pH, density, refractive index, surface tension, viscosity, conductivity, and polarity) after a freeze-drying process. Then, cork extraction was conducted for each NaDES in a high-pressure closed system, leading to three NaDES-based samples (Extract 1–3) with antioxidant and antibacterial properties. Once assessed with regard to their antioxidant activity, transdermal permeability, and cytotoxicity, all samples were added to two commercial cosmetic products. The new complexes (Formulation A and Formulation B) showed an enhanced antioxidant activity and no cytotoxicity on keratinocytes (for extract concentrations up to 10 mg/mL). Furthermore, Rocha et al. suggested that Extract 2 (corresponding to NADES 2 solvent) would be the most suitable for inclusion in cosmetic formulations.

Marijan et al. [91] conducted an extraction using NaDES to derive bioactive compounds from flowering aerial parts of *Lotus corniculatus* (*LC*), *Medicago lupulina* (*ML*), and *Knautia arvensis* (*KA*), as well as from leaves of *Plantago major* (*PM*) selected from urban parks. In this work, the authors demonstrated that organic waste from urban areas can contain useful minerals for skin health. The UA extraction was exploited using an NaDES composed by glycerol, betaine and glucose (in a weight ratio of 20:4:1) and then diluted with water in the proportion 8:2 (DES80) or 4:6 (DES40) in order to investigate two different polarities of the solvent [17,30]. Furthermore, for a comparative evaluation, Marijan et al. utilized environmentally friendly extraction solvents made by dissolving hydroxypropyl-β-cyclodextrin (HPβCD) or γ-cyclodextrin (γCD) in aqueous solutions. The -different metals identified by extraction in plants were calcium, very abundant in *PM*, zinc, especially present in *KA*, iron, in *ML* and less in *LC*. The last two extracts (obtained by DES80) were the richest in phenols; in *ML* samples, the authors identified quercetin, kaempferol, luteolin and apigenin, while in *LC* samples, only kaempferol was detected. Differently, in *KA* and *PM* extracts, the highest concentration of phenols was obtained from DES40. In particular, in *PM*, all of the studied phenols except luteolin were identified, while in *KA,* only apigenin and luteolin were detected. The results obtained by Marijan et al. seem to indicate *LC extract* as a good anti-tyrosinase agent and *KA* as a better anti-elastase one. In conclusion, the solvents they used, in addition to contributing to bioactivity, allowed for the recovery of bioactive compounds and metals in organic waste from urban parks, which can be exploited to produce ecological cosmetic formulations with added high value.

Another research group, Alishlah et al. [92], optimized a UA extraction of oxyresveratrol from the root powder of *Morus alba* (mulberry) urea and glycerin eutectic systems. The aim of this study was the formulation of an efficient skin whitening cosmetic lotion containing the NaDES-based extracts. The heating and stirring method was selected for the preparation and evaluation of NaDES with a urea-glycerin molar ratio of 1:1, 1:2, and 1:3. UAE was performed with different extraction times (i.e., 10, 15 and 20 min) using 20 mL NaDES for 1 g mulberry powder; HPLC was used for the identification and quantitation of oxyresveratrol after extraction. The best results in terms of oxyresveratrol recovery (2.42 mg/g dry powder) were obtained in 15 min by NaDES with urea-glycerin 1:3. Therefore, this sample was used at a percentage of 35% *w*/*w* to formulate three oil-in-water emulsions (whitening skin lotions: formula A, formula B and formula C) containing stearic acid (1%), isopropyl myristate (5%), propylene glycol (15%) cetyl alcohol (2% A, 4% B, or 6%C), Tween 80 (3.88% A, 4.13% B, or 4.29% C) and glyceryl monostearate (1.12% A, 0.87% B, or 0.71% C). Based on physical evaluations, formula A was chosen as the best formulation for the development of a cosmetic bleaching product for the skin.

Oktaviyanti et al. [93] developed and optimized a green ultrasound-assisted deep eutectic solvent extraction of *Ixora javanica* flowers to obtain a natural antioxidant and skin lightening agent to be used in the cosmetic field. The researchers evaluated the extraction efficiency of 11 NaDES regarding flavonoids and anthocyanins, and the antioxidant and anti-tyrosinase activity of the obtained extracts. By use of the heating method, choline chloride (Ch) was coupled in opportune molar ratios with different HBDs (polyols and organic acids) to form the following eutectic systems: ChPg (choline-propylene glycol 1:1), ChGl (choline-glycerol 1:2), ChEg (choline-ethylene glycol 1:2), ChPeg (choline-polyethylene glycol 1:2), ChSb (choline-sorbitol 1:1), ChPd (choline-1,3-propanediol 1:3), ChOa (choline-oxalic acid 1:1), ChLa (choline-lactic acid 1:2), ChGa (choline-glycolic acid 1:2), ChMa (choline-malic acid 1:1) and ChCa (choline-citric acid 1:1). According to the authors, the best extractive NaDES solvent for *I. javanica* flowers was ChPg, which demonstrated the best-performing anti-tyrosinase activity. The design and optimization of the extraction parameters to maximize flavonoids recovery was achieved by the response surface methodology (RSM); the best-performing conditions required an extraction temperature at 57 °C for 5 min with a matrix-solvent ratio of 1:50 g/mL. The authors concluded that NaDES can be used as useful green alternative organic solvents for bioactive compound extractions from natural models to be added in cosmetic formulations.

In addition to natural models, agrifood waste also represents a promising renewable source of bioactive cosmetic ingredients. In this regard, Punzo et al. [94] studied NaDES for the extraction of polyphenols, from freeze-dried red grape pomace, for topical applications. NaDES, prepared by the heating and stirring method, were obtained by three HBDs (urea, citric acid and ethylene glycol), selected on the basis of their proven skin compatibility and mixed in optimal molar ratio with betaine (HBA), as follows: betaine-citric acid 1:1 (BET-CA), betaine-ethylene glycol 1:2 (BET-EG), and betaine-urea 1:2 (BET-U). Among the samples obtained and used directly as topical formulations, BET-CA extracts (the richest in malvidin), showed the best antioxidant and anti-inflammatory activity at concentrations able to permeate the skin. Therefore, this formulation was indicated by Punzo et al. as the most suitable ingredient for anti-aging cosmetic formulations. Moreover, NaDES were proven as excellent extractants and carriers for polyphenols; the researchers assessed and confirmed the in vitro safety of NaDES extracts by means of human 3D keratinocytes. The authors concluded that their findings could support the use of NaDES as promising cosmetic ingredients and carriers in new drug delivery systems for topical applications, since they can affect the permeation of active molecules.

## 3. NADES in the Pharmaceutical Field

As stated in the reported literature, NaDES are not only considered as green alternatives to conventional organic solvents, but they also promote and enhance the extraction of bioactive compounds from natural models, or agrifood waste, suitable for cosmetic application. More recently, several publications have referred to the exploitation of NaDES in pharmaceutical technology to solubilize and stabilize a wide range of pharmaceutical systems. Moreover, they can be applied in hydrogels and film formation and as carriers to deliver bioactive compounds in many other innovative pharmaceutical forms.

In this regard, Delgado-Rangel et al. [95] used NaDES, without crosslinkers, to create 3D pure and porous materials constituted by chitosan (CTS), to be used against *V. cholerae* biofilm. In particular, the research group optimized an environmentally friendly method that allowed the formation of porous monoliths and films, underlining the versatility of application of NaDES-assisted phase separation processes. The preparation of CTS matrix films was carried out in three steps by evaporation-induced phase separation. The solution, based on 2% CTS and acetic acid, was mixed with a NaDES obtained by the heating method and composed by a mixture of chloride choline-urea in a molar ratio of 1:2 (CCU-DES). After the evaporation of the acidic aqueous solvent from the CTS CCU-DES mixture, the plasticized CTS film structure was obtained. Between the different weight ratios of CTS/CUU analyzed, Delgado-Rangel et al. selected the equal weight ratio, as it allowed obtaining films with the most suitable porous structure. In addition, the research group observed that the thermal stability of CTS was affected by residual NaDES within its porous structure. They concluded that, as regards films formed by chitosan in this specific example, the porosity influenced *V. cholerae* growth. 

Differently, the research group of Alkhawaja et al. [96] used NaDES as a carrier of a phosphodiesterase 5 (PDE-5) enzyme inhibitor, namely tadalafil (TDF), with the aim of developing a formulation to be applied on burns and able to prevent the systemic absorption of the drug. By stirring at room temperature, the authors prepared seven NaDES formulations, based on malonic acid (MA) and choline chloride (CC) in different molar ratios, with and without propylene glycol (PG) to provide different viscosities. B01, B02, and B03 formulations were composed by the simple mixture of MA-CC 1:1, 1:2, and 2:1, respectively. Once prepared, these NaDES were mixed with PG at different ratios to obtain the B04 formulation (B01-PG 1:1), B05 formulation (B01-PG 1:2), B06 formulation (B02-PG 1:1), and B07 formulation (B02-PG 1:2). The characterization of blank NaDES, obtained by evaluation of spreadability and measurement of contact angle, allowed the selection of B01 and B04 as potential topical formulations. Subsequently, Alkhawaja and co-workers improved the aqueous solubility of TDF by mixing it into B01. Moreover, a new formulation (F01) was developed by incorporating lidocaine (LCD) into the NaDES samples containing TFD, to also provide a local anesthetic effect. Due to topical effects, B01 and B04 were chosen to formulate topical preparations with or without lidocaine. The authors concluded that F01 delays the healing process, thereby lowering the probability of scarring that may result from burn wounds. Moreover, the presence of NaDES in the formulations, having antimicrobial activities, reduces the risk of bacterial infections.

Filip et al. [97] coupled NaDES with hydroxypropylcellulose (HPC) to produce self-assembled hydrogels (HPC-NaDES), compatible with the human gingival fibroblast (HGF) cell line, for applications in the pharmaceutical field. In particular, the authors obtained HPC-NaDES 17% and HPC-NaDES 29% aqueous solutions by adding to a 14% HPC solution the NaDES previously prepared. Choline chloride (ChCl) was mixed in different molar ratios with four HBDs and small water amounts to obtain ChCl-urea 1:2 (U), ChCl-glycerol 1:2 (GL), ChCl-lactic acid 1:1 (LA), and ChCl-citric acid 1:1 (CA). HPC-NaDES were then characterized by FT-IR, H^1^NMR, DSC, TGA measurements, and rheological tests. According to the authors, the HPC-NaDES physicochemical properties are influenced by different parameters such as hydrogen bond interactions between HBA and HBD, content of NaDES, and the water amount. Stronger hydrogen bonds were observed in HPC-CA and HPC-GL compared to the other hydrogels obtained. All HPC-NaDES hydrogels exhibited a pseudoplastic behavior. Furthermore, the latter possessed thermo-thickening characteristics since the HPC in aqueous systems has a lower critical temperature than the solution itself. Finally, the disk diffusion methods [98,99] enabled the determination of antibacterial and antifungal activities, showing this order of efficacy: ChCl-CA > ChCl-LA > ChCl-U > ChCl-GL.

A research team that exploited the solubilizing abilities of NaDES (Mustafa et al. [100]) screened various types of eutectic mixtures to solubilize poorly water-soluble drugs and produce liquid formulations for parenteral administration and gastric tube feeding. In particular, the authors conducted tests of NaDES solubilization on some insoluble drugs such as nitrofurantoin, trimethoprim, griseofulvin, methylphenidate, and spironolactone, and on water-unstable ones (trichloroacetaldehyde monohydrate or chloral hydrate). They observed good drug solubility in eutectic systems based on choline chloride or betaine, coupled with different HBDs, such as organic acids, sugars, and polyols. Good results were achieved for methylphenidate, trimethoprim, griseofulvin, spironolactone, and nitrofurantoin. In addition, the stability of NaDES samples containing drugs was tested at 4 °C for up to 4 months. The results suggested that methylphenidate and trimethoprim are better solubilized in acidic NaDES, while pure acetic or lactic acids are more effective for spironolactone and griseofulvin solubilization. Nitrofurantoin could be dissolved only by a mixture of choline chloride–acetic-acid–proline–water (1:1:1:5 molar ratio) at a concentration of 5 mg/mL. Unstable drugs dissolved at the maximum concentration of 250 mg/mL. Therefore, Mustafa et al. suggested that NaDES represent promising solvents to optimize liquid formulations with poorly water-soluble drugs, but further investigations are needed. 

Li et al. [101] exploited the features of NaDES with the aim of improving the antibacterial properties of a hydrogel to be used as a wound dressing. Specifically, they prepared a hydrogel of sodium hyaluronate (SH), coated with dopamine (DA), using a NaDES composed by choline chloride and glucose. After combining SH and DA, Li et al. added N-Hydroxysuccinimide (NHS) and 1-ethyl-3-(3-(dimethylamino)propyl) carbodiimide (EDC) as coupling agents to the mixture using the techniques described by Lee et al. [102]. The resulting product, i.e., the SH conjugate with dopamine (DASH), was then purified and lyophilized. Then, DASH and NaDES were combined in a mass ratio of 4:175 to form a DES-DASH hydrogel. Subsequently, a DES-DASH@Ag hydrogel containing a silver nitrate solution was prepared and tested for its antibacterial activity against *S. aureus* and *E. coli*. The results showed a nontoxic behavior towards NIH-3 T3 fibroblast cell lines and the ability to support wound healing in mouse skin within 12 days of surgery. Thus, Li et al. suggested a future use of the DES-DASH@Ag hydrogel as a topical application for wound dressing.

Sokolova et al. [103] exploited the plasticizing effect of chitosan of (CS) with NaDES to create CS/DES films. According to the method described by Samarov et al. [104], they prepared NaDES by mixing malonic acid (MA) with choline chloride (ChCl). The CS/DES films (with a thickness of 20 µm) were obtained by casting at room temperature a mixture of CS and water with a NaDES content ranging from 0 to 82% (*w*/*w*), in Petri disks. Film characterization was performed by means of Fourier transform infrared spectroscopy (FT-IR), scanning electron microscopy (SEM), atomic force microscopy (AFM), water absorption isotherms, mechanical measurements, thermogravimetric analysis (TA) and differential scanning calorimetry (DSC). The analysis of water absorption isotherms, AFM data, and FTIR spectra indicated that during NaDES formation, MA and ChCl strongly interact, as well as CS and DES. The other results obtained by Sokolova et al. indicated a glass transition temperature between +2.0 and −2.3 °C, with maximum elongation at break of 62% shown by a film containing 67% by weight of NaDES. The increase in NaDES content (from 0 to 82%) led to a decrease in elasticity at tension from 800 MPa down to 16 MPa. Additionally, film with 82% NaDES demonstrated an elastic modulus with a bimodal trend. Finally, all of the studied films were found to be soluble in water at room temperature.

A further paper, in which NADES were used to endow plasticizing properties to chitosan films, was presented by Pontillo et al. [105]. The authors investigated the solubility of chitosan in NaDES aqueous solutions of choline chloride: lactic acid (ChCl-LA, molar ratio 1:1.5) and betaine: lactic acid (bet-LA, 1:2), demonstrating that chitosan can be dissolved in 1% NaDES *w*/*v* water solutions. Films prepared by the casting technique were compact, with elasticity properties comparable to films obtained by chitosan dissolved in 1% acetic acid (F/AA). Films containing NaDES or physical mixtures were more elastic and pliable. Films prepared with NaDES solutions (F/bet:LA NaDES and F/ChCl:LA NaDES) were significantly thicker than the F/AA films. The influence of acids on films’mechanical properties was confirmed by comparing different organic acids: the presence of lactic acid instead of acetic acid increases both the resistance of burst and the maximum elongation of chitosan films. Interesting results were obtained observing SEM morphology: F/AA films presented small holes, possibly due to the evaporation of the acetic acid, while the two F/bet:LA NaDES and F/ChCl:LA NaDES seemed to have a smooth surface with small wrinkled structured pores. The results suggested that properties of the films can be easily adjusted to fit the requirements useful for a wide range of applications; in particular, the new mixtures could represent promising alternatives for biomedical wound healing patches that usually lack in terms of elasticity.

Cerdá-Bernard et al. [106] investigated chitosan/alginate hydrogels to stabilize freeze-dried *C. sativus* flower extracts obtained by NaDES-UAE. The study aim was to exploit unused flower waste, reducing its environmental impact while stabilizing high added-value ingredients to screen their potential practical applications. In particular, they proposed an innovative extraction method that afforded the preparation of stable bioactive compounds with excellent antioxidant activity. NaDES obtained by the heating method [107] were based on different molar ratios of betaine-lactic acid (80%)-water 1:2:2.5 (Bet/LA/Water), glucose-lactic acid (80%)-water 1:5:6.2 (Glu/LA/Water), betaine-glycerol-water 1:3:1 (Bet/Gly/Water), L-proline-citric acid-water 2:1:3 (Pro/CA/Water), and L-proline-glycerol 1:2 (Pro/Gly), this last proving to be the best sample. The optimal UAE parameters for the extraction of saffron floral by-products and stigmas considered a process time of 20 min at 180 W and 90% Pro/Gly. Showing excellent antioxidant properties, these extracts were subsequently incorporated into a 0.3% chitosan/2% alginate hydrogel for stability improvement and to study their possible application as food formulations. Hydrogels containing extracts were then evaluated with regard to their water uptake and water retention capacities and total phenolic content (TPC) during in vitro digestion. Hydrogels with saffron stigma NaDES extracts showed an increased TPC after an hour of intestinal digestion, with constant levels up to 2 h. Otherwise, the hydrogel with saffron floral by-product NaDES extract showed an increase in TPC within the first 2 h. Therefore, Cerdá et al. proposed NaDES-UAE as an optimal combination for the recovery of bioactive compounds from saffron flower waste and suggested possible uses of their hydrogels for the cosmetic, food and pharmaceutical areas.

Silva et al. [108] presented a drug delivery system in which curcumin, dissolved in NaDES based on choline chloride (CC) and glycerol (GLY), was encapsulated into beads obtained by ionotropic gelation with chitosan and alginate. Beads were produced using an extrusion-dripping method. The main goal of the study was to develop curcumin-loaded hydrogel beads with an improved solubility and stability during transit along the gastrointestinal tract. In this context, NaDES can offer a green and promising alternative to overcome solubility hurdles and the need for removing organic solvent.

Wang et al. [109] investigated a hydrogel entirely composed by natural ingredients (sodium hyaluronate—SH, dopamine—DA, chitosan—CS, aloe vera—AV and NaDES) to be applied as a green and degradable wound dressing formulation. The hydrogel showed good cytocompatibility on NIH-3T3 fibroblast cells, and antibacterial properties against both Gram-positive (*S. aureus*) and Gram-negative (*E. coli*) bacteria. Sample surface morphologies were characterized by scanning electron microscopy (SEM); hydrogel swelling and in vitro degradation studies were assessed by mass change in phosphate buffered saline (PBS) solutions at 37 °C, and the dynamic rheological performances were evaluated by a strain-controlled rheometer. The results obtained by the NaDES-SH-CS/DA/AV hydrogel showed good cytocompatibility on NIH-3T3 fibroblast cells and good antibacterial properties. Moreover, the formulation promoted skin tissue regeneration with good wound healing effects on mouse skin within 12 days of surgery. 

A new approach to NaDES application in the pharmaceutical and cosmetic fields seems to be exploitable by transforming these natural solvents into eutectogels for active ingredient delivery. Zeng et al. [110] proposed this opportunity using xanthan gum, a well-known polysaccharidic gelling agent produced by bacterial fermentation. This low-cost, biocompatible and biodegradable polymeric excipient is widely used in hydrogel preparations for biomedical and technological applications. Recently, it attracted a great deal of attention as a biomaterial for tissue scaffold preparation (extracellular matrix) in tissue engineering studies. The authors prepared highly biodegradable, thermostable eutectogels, by gelation with xanthan gum, of four low-viscosity NaDES containing choline chloride as the HBA and glycerol, xylitol, sorbitol or citric acid as the HBD. Gelation was obtained at low concentrations of xanthan gum (less than 5%). Morphology of the xanthan gum eutectogels was observed by optical and electron microscopy, and the possible gel formation mechanism was investigated by Fourier-transform infrared spectroscopy (FT-IR) and X-ray diffraction (XRD). The rheological properties were also studied, and the results showed excellent thermostability of the eutectogels in a temperature range of from 60 to 80 °C, with unchanged weight, keeping the gel stored at 80 °C for 10 h. By comparison with xanthan gum hydrogels, the resulting eutectogels were more stable in response to temperature increases, providing good rheological characteristics that were maintained over time.

## 4. Conclusions

In this review, the potential of NaDES as alternative green solvents in the extraction of natural active ingredients and as drug carriers was presented and explored. The most significant papers of the last 5 years regarding cosmetic and pharmaceutical formulations were discussed.

The main difficulties in the application of NaDES in industrial extraction processes are often represented by the high viscosities and by the separation of the solute after extraction. This second drawback is usually overcome by water addition or increasing the temperature, since these substances are thermo and pH switchable.

This review could help to disclose new opportunities and challenges for NaDES implementation not only as green alternative solvents but also as potential useful pathways to deliver bioactive ingredients in innovative formulations.

In conclusion, their application versatility, safety, biodegradability, biocompatibility and natural origin support NaDES as solvents of the future in the food, cosmetic and pharmaceutical fields.

## Figures and Tables

**Table 1:** Extraction of target compounds from natural sources and agri-food byproducts using NADES as alternatives to conventional solvents, and related references.

Target Compound	Natural Matrix	NaDES System	Conventional Solvent	Reference
Anthocyanins	Grape skin	Citric acid/D-(+)-maltose	water or organic solvents such as methanol and ethanol	[34]
Anthocyanins	Mulberry	Choline chloride/citric acid/glucose	methanol, ethanol, acetic acid modified water or hydrochloric acid modified ethanol	[35]
Anthocyanins	Grape pomace	Choline chloride/citric acid Choline chloride/proline/malic acid	methanol, acetone and hydrochloric acid	[36]
Anthocyanins	Sour cherry pomace	Choline chloride/malic acid	acidified ethanol	[37]
Anthocyanins	Blueberry peel	Choline chloride/malic acid Choline chloride/citric acid	acidified ethanol	[38]
Caffeine	Chinese dark tea	Choline chloride/lactic acid	chloroform, dichloromethane, acetone and ethyl acetate.	[39]
Curcumin	Standard solubility tests	Choline chloride/glycine	ethanol, methanol, acetone and ethyl acetate	[40]
Hydroxytyrosol	Olive leaves	Citric acid/glycine/water	ethanol and water	[41]
Isoflavones	Soybeans	Choline chloride/citric acid	acetonitrile acetone, ethanol and methanol	[42]
Pectins	Mango peel	Betaine/citric acid choline chloride/malic acid	alkaline, acidic aqueous solutions and enzyme	[43]
Phenolic acids	Orange peel	Choline chloride/ D-(+)-glucose/water	acetonitrile, methanol and acetone	[44]
Phenolic compounds	Bitter melon	Choline chloride/acetic acid	ethanol, methanol, acetone, ethyl acetate and chloroform	[45]
Phenolic compounds	*Olea europaea*	Water/Choline chloride/fructose	dimethyl sulfoxide, hexane, ethanol and methanol	[46]
Phenolic compounds	Olive pomace	Choline chloride/citric acid	petroleum ether, acetone, ethyl acetate and methanol	[47]
Phenolic compounds	Hazelnut skin	Choline chloride/lactic acid	methanol, ethanol, and methanol/water mixtures	[48]
Phenolic compounds	Cocoa beans	Betaine/glucose	Hexane, petroleum ether, methanol, ethanol, ethyl acetate and acetone	[49]
Phenolic compounds	Waste mango peel	Lactic acid/glucose	methanol, ethanol, acetone and ethyl acetate	[50]
Rosmarinic acid, carnosol, carnosic acid	*Rosmarinus officinalis*	Lactic acid-glucose/menthol-lauric acid (biphasic system)	dichloromethane, ethanol and methanol	[51]
Solenesol	Tobacco leaves	Choline chloride/urea	petroleum ether, acetone, *n*-hexane, ethyl acetate and methanol	[52]
Tryptanthrin, indirubin, and indigo	*Baphicacanthus cusia*	Lactic acid/L-menthol	methanol, ethanol and methanol/dichloromethane	[53]
Triterpenic acids, Ursolic acid	* Eucalyptus globulus *	Choline chloride/D-(+)-glucose	dichloromethane *n*-hexane ethanol or chloroform	[54]

## Data Availability

Not applicable.
